# Prognostic factors in radiotherapy of anaplastic thyroid carcinoma: a single center study over 31 years

**DOI:** 10.1186/s13014-023-02249-w

**Published:** 2023-04-19

**Authors:** Julia Jacob, Dirk Vordermark, Kerstin Lorenz, Daniel Medenwald

**Affiliations:** 1 Martin Luther University Halle-Wittenberg, 06120 Halle, Germany; 2 Department of Radiation Oncology, Martin Luther University Halle-Wittenberg, 06120 Halle, Germany; 3 Department of Visceral-, Vascular, and Endocrine Surgery, Martin Luther University Halle-Wittenberg, 06120 Halle, Germany

**Keywords:** Anaplastic thyroid cancer (ATC), Radiotherapy, Prognostic factors, Multimodal therapy, Long-term-study

## Abstract

**Background:**

Anaplastic thyroid carcinoma has a very poor prognosis. We analyzed the effect of surgery, radiotherapy and chemotherapy on survival time and side effects in patients with ATC.

**Methods:**

We retrospectively analyzed all patients (n = 63) with histologically confirmed ATC who presented at our clinic between 1989 and 2020. We analyzed the survival with Kaplan–Meier curves and cox proportional hazard models and acute toxicities with logistic regression models.

**Results:**

Out of 63 patients, 62 received radiotherapy, 74% underwent surgery and 24% received combined chemotherapy. A median radiation dose of 49 Gy (range 4–66 Gy) was applied. In 32% of the cases opposing-field technique was used, in 18% 3D-conformal, in 27% a combination of opposing field and 3D-conformal technique and 21% obtained IMRT (intensity modulated radiotherapy) or VMAT (volumetric modulated arc radiotherapy). Median overall survival (OS) was 6 months. We identified five predictive factors relevant for survival: absence of distant metastases at the time of diagnosis (OS 8 months), surgery (OS 9.8 months), resection status R0 (OS 14 months), radiation dose of 50 Gy or higher (OS 13 months) and multimodal therapy (surgery, radiotherapy and chemotherapy) with a median OS of 9.7 months.

**Conclusion:**

In spite of the dismal outcome, longer survival can be achieved in some patients with ATC using surgery and radiotherapy with a high radiation dose. Compared to our previous study, there are no significant advantages in overall survival.

*Trial registration* Retrospectively registered.

## Background

Anaplastic thyroid carcinoma (ATC) is a rare but aggressive neoplasia. It represents only 1–2% of all thyroid malignancies but causes half of the deaths in thyroid carcinoma [1]. Whereas other thyroid cancers in comparison have a good prognosis, the outcome of ATC is very poor. The median survival of ATC is about five months [2]. Because of its aggressive characteristics, it shows an invasive and fast growth into surrounding structures such as the trachea, esophagus, and muscles [3]. Hence, it causes dysphagia and dyspnea and often surgery cannot achieve satisfying outcomes [4]. Furthermore, it is often metastasized at the time of diagnosis, mostly into lungs and pleura [3]. Additionally, radioiodine uptake in ATC is negligible due to the loss of the ability to take up I-131 and shows radio resistance [5]. Due to the poor response to conventional therapies, the therapeutic approaches have changed in recent years. With the availability of molecular testing, targeted therapies have been developed [3]. Also, there have been new developments in radiotherapy: IMRT and VMAT are now the main techniques which allow the application of a higher radiation dose and less side effects [3].

Due to the small number of cases and short survival time, there are very few randomized studies for ATC treatment. Thus, treatment recommendations must largely be based on non-randomized trails. The current study is an update on an earlier publication by our group [6]. Following that study, we continued the research with a greater number of cases over a longer period.

## Methods

We performed a retrospective analysis of all patients treated for histologically proven ATC at the Department of Radiation Oncology, Martin Luther University Halle-Wittenberg between 1989 and 2020. Information about the tumor and therapy were extracted from patient records. Information on survival was obtained from cancer registries, the registration office and the discretion of the treating general physician. The retrospective analysis was performed in compliance with the local ethics commission of the Medical Faculty, Martin Luther University Halle-Wittenberg, no. 2020-078.

### Statistic analysis

We used univariate and multivariate analyses. For univariate survival analysis we computed Kaplan-Meyer-curves. In analyses of overall survival (from diagnosis to death), we compared the groups with a log-rank test. Results with a p-value lower than 0.05 were regarded as statistically significant. Patients who were alive at last follow-up were censored. We also performed uni- and multivariate analyses applying Cox proportional hazard models estimating hazard ratios and 95% confidence intervals to screen the efficiency of the major treatment modalities. By building different models, we tried to adjust for the confounders age, gender and Karnofsky performance score and compared all therapy options in one model, adjusted for these confounders. For evaluating the side effects of radiation, we used logistic regression models. The analysis of data was performed with the statistic program SPSS version 26.

## Results

Characteristics of enrolled patients are presented in Table 1. We observed 63 patients of whom 37 (59%) were female and 26 (41%) were male. The median age at diagnosis was 68 years with a range from 38 to 88 years.

**Table 1:** Patients baseline characteristics (n = 63)

	n	Percentage
*Median age* (in years, range)		68 (38–88)
*Sex*		
Female	37	59%
Male	26	41%
*N stage*		
N0	13	21% (32.5%)*
N +	27	43% (67.5%)*
NX	23	36%
*M stage*		
M0	32	56%
M1 (at diagnosis)	28	44%
*UICC stage*		
IVA	1	2%
IVB	34	54%
IVC	28	44%
*Karnofsky performance status*	20	Median: 60
*Any surgery*	48	76%
Total thyroidectomy	33	52%
Subtotal thyroidectomy	10	16%
Hemithyroidectomy	5	8%
Removal/Dissection of lymph nodes	28	44%
*Resection status*		
R0	6	12.50%
R1	6	12.50%
R2	28	58%
RX	11	17%
*Molecular testing*	2	3%
BRAF, MEK	1	1.50%
*TP95*	1	1.50%
*Targeted therapy*	3	5%
*Chemotherapy*	15	24%
Radiochemotherapy (concurrent)	11	17%
Induction chemotherapy	2	3%
Chemotherapy after radiotherapy)	2	3%
	7	
(Radio)chemotherapy (patient with M1)	6	11%
(Radio)chemotherapy (patients with M0)	2	9.50%
(Radio)chemotherapy for patients with no reported M-stage		3%
*Radiotherapy*	62	98%
Opposing field technique	20	32%
3D-conformal technique	11	18%
Combination of opposing field and 3D-conformal technique	17	27%
IMRT	9	15%
VMAT	4	6%
unknown	1	2%
Median applied dose (Gy, range)		41.5 (4–66)
*Histology*		
Pure ATC	45	71%
ATC + differentiated component	18	29%

* percentage for patients with reported N-stage (n = 40). IMRT = Intensity modulated radiotherapy. VMAT = Volumetric modulated arc therapy. ATC = Anaplastic thyroid cancer

All cases had UICC-status IV (8th edition) [20]. 2% had status IVA, 54% IVB and 44% IVC.

The Karnofsky performance status was reported for 20 patients and was 60 in the median.

We had data about lymph node involvement for 40 patients, from which 67.5% had effected lymph nodes and 32.5% had N0.

Distant metastases were found in 44% at the time of diagnosis and in 41% in the course of treatment. The main localization of metastasis were the lungs in 63% of all cases, bones (21%), central nervous system (14%), extracervical lymph nodes (13%) and liver (10%).

Recurrent laryngeal nerve palsy the time of radiotherapy was reported for 31 cases (49%). Out of all patients, 76% underwent surgery (52% total, 16% subtotal thyroidectomy, 8% hemithyroidectomy). In 12.5% R0 (no margins) was achieved. A resection status of R1 was reached in 12.5% and R2 in 58%. In 17% the resection status could not be determined.

Molecular Testing was reported for two patients. One was BRAF and MEK negative and received Lenvatinib and Pembrolizumab. The other patient was TP53 positive, this did not lead to any targeted therapy.

Targeted therapy was given to three patients. They received Pembrolizumab and Lenvatinib.

Chemotherapy was administered to 15 patients (24%), of which 11 (17%) got simultaneous radiotherapy. In detail, (radio)chemotherapy was given to seven patients with disseminated disease. Six patients had no metastases and received curative (radio)chemotherapy. The most frequently used chemotherapeutics were Carboplatin, Cisplatin, Paclitaxel and Doxorubicin, in mono- or combined therapy. Radiotherapy (RT) was delivered to 62 patients; one refused the radiation. 32% of the patients were irradiated via opposing-field technique, 18% with 3D-conformal technique and 27% with a combination of both. In 15%, volumetric modulated arc therapy (VMAT) and in 6%, intensity modulated radiation therapy (IMRT) technique was applied. The median total dose was 49 Gy with a range from 4 to 66 Gy. In ten patients (16%) the radiotherapy had to be terminated prematurely due to a deterioration of health condition or death. For 15 patients (24%) a tracheostomy was performed. We described toxic side effects using grades according to the common terminology criteria of adverse events (CTCAE), version 5.0 [7]. Dysphagia caused by radiation appeared in grade 0, 1, 2, 3, 4 or 5 in 3%, 27%, 22%, 25%, 16% and 2%, respectively (no report in 5%). For adequate nutrition 18 patients (29%) had to be fed by parental nutrition or tube, placed during the course of therapy or prophylactically before initiation.

Skin toxicity was reported in CTCAE grade 1, 2 and 3 in 21%, 37% and 18%. Seven patients (11%) had no erythema and for eight it was not reported.

### Survival analyses

**Fig. 1:**
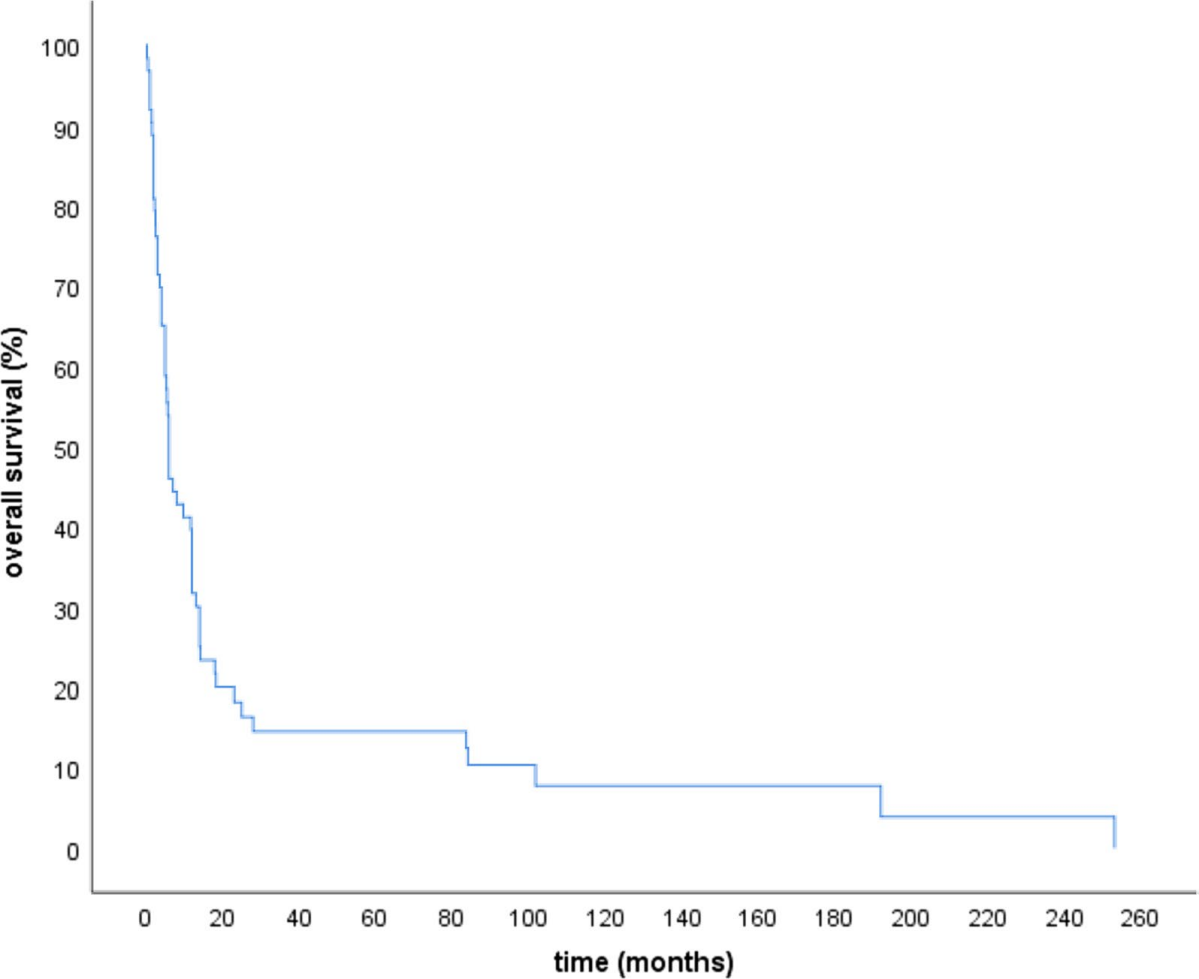
Overall survival for all patients with ATC (n = 63)

**Fig. 2:**
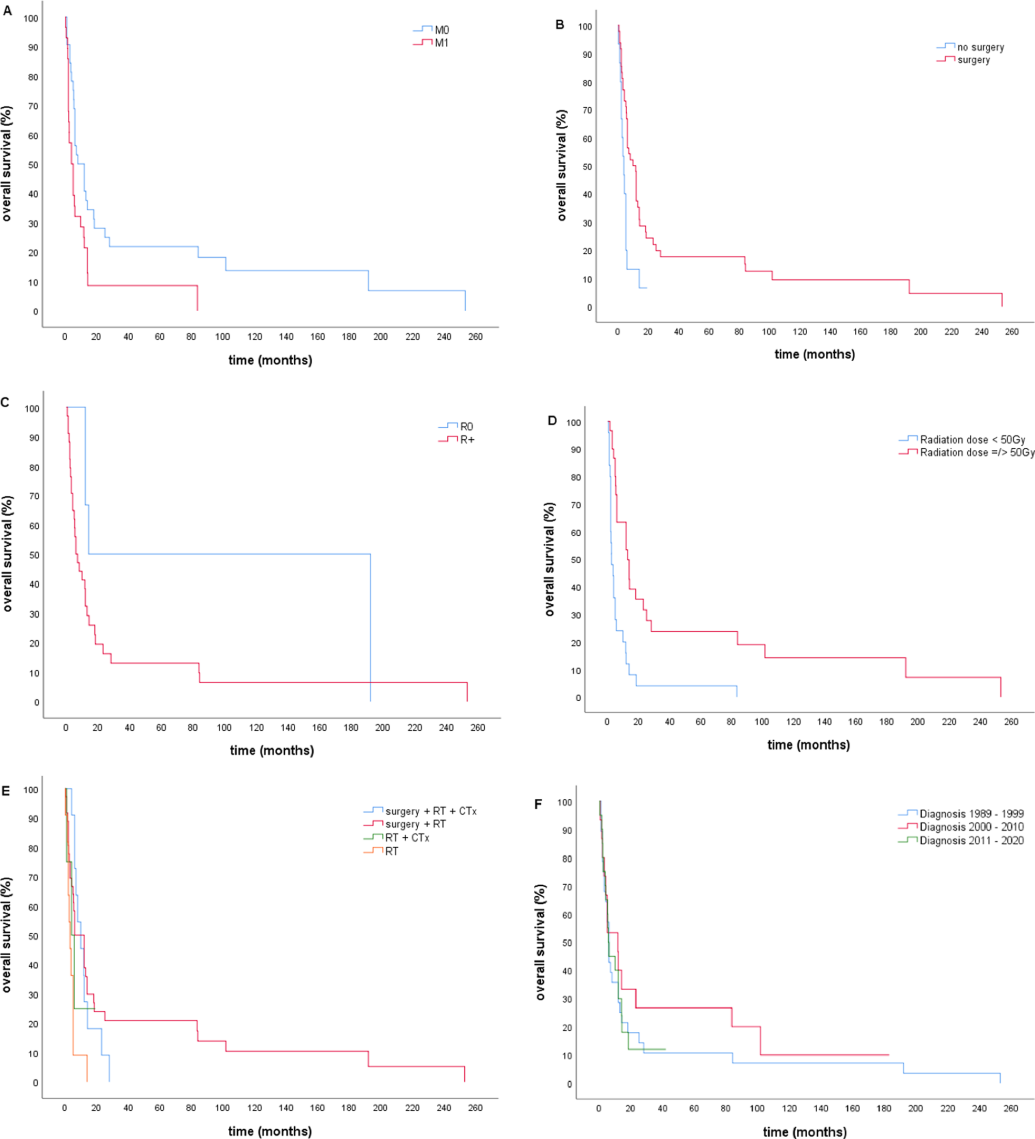
Overall survival for: **A** patients with M0 vs. M1 **B** patients who underwent surgery vs. no surgery **C** patients with R0 vs. R + **D** patients who received a radiation dose of at least 50 Gy vs. lower than 50 Gy **E** patients who received surgery + radiotherapy (RT) + chemotherapy (CTx) versus surgery + radiotherapy versus radiotherapy + chemotherapy versus only radiotherapy **F** patients diagnosed with ATC between 1989 and 1999 versus 2000 and 2009 versus 2010 and 2020

The median overall survival (OS) was 6 months and 30% lived longer than one year (Table 2, Fig. 1). Ten patients (16%) lived longer than 24 months and three (5%) longer than 60 months (5 years). Significant predictors for longer survival were the absence of distant metastases at the time of diagnosis (HR = 0.5 95% CI: 0.3–0.9, surgery (HR = 0.4, 95% CI: 0.2–0.8), complete resection (R0) (HR = 0.4, 95% CI: 0.1–1.0) a total radiation dose of 50 Gy or higher (HR = 0.3, 95% CI: 0.2–0.5) and the use of multimodal therapy (HR = 0.4, 95% CI: 0.1–0.8) (Fig. 2).

**Table 2:** Univariate survival analysis using Kaplan–Meier-curves and Cox proportional hazard models

Parameter	Subgroups	n	Median OS (months)	*p*	HR (95% CI)
All patients			6.0		
Age	< 67 years	24	12.0	0.074	0.6 (0.4–1.1)
	≥ 67 years	39	5.0		1
Sex	Female	37	5.0	0.892	1.0 (0.6–1.6)
	Male	26	7.0		1
N Stage	N0	13	14.0	0.088	0.5(0.3–1.1)
	N +	27	6.0		1
M Stage	M0	32	8.0	**0.008**	**0.5 (0.3–0.9)**
	M +	28	4.0		**1**
Surgery	Yes	48	9.8	**0.003**	**0.4 (0.2–0.8)**
	No	15	3.6		**1**
Resection status	R0	6	14.0	**0.047**	**0.4 (0.1–1.0)**
	R1, R2	34	6.0		**1**
Lymph node dissection	Yes	28	12.0	0.317	0.8 (0.4–1.3)
	No	35	5.0		1
Radiation dose	< 50 Gy	31	2.5	** < 0.0001**	**0.3 (0.2–0.5)**
	≥ 50 Gy	31	13.0		**1**
Radiation delivery technique	Opposing field/ 3D	49	6.0	0.52	0.8 (0.4–1.6)
	IMRT/VMAT	13	9.8		1
Chemotherapy	Yes	15	8.0	0.946	1.0 (0.6–1.9)
	No	48	5.3		1
Chemotherapy for patients with M0	Yes	6	6.0	0.246	1.697 (0.7–4.307 =
	No	24	12.0		1
Chemotherapy for patients with M1	Yes	7	10.0	0.222	0.5 (0.2–1.4)
	No	21	3.0		1
Combined	Yes	11	9.8	0.908	1.0 (0.5–1.9)
Radiochemotherapy	No	4	5.5		1
Histology	Pure ATC	45	9.8	0.731	0.9 (0.5–1.6)
	ATC + DTC	18	6.0		1
Therapy modality	Surgery + RT + CTx	11	9.8	**0.006**	**0.4 (0.1–0.8)**
	Surgery + RT	36	6.0		**0.3 (0.1–0.6)**
	RT + CTx	4	4.0		0.4 (0.1 -1.5)
	Rt alone	11	3.0		1
	(Surgery alone)	1	(censored)		
Year of diagnosis	1989–1999	28	6.0	0.667	1
	2000–2009	15	11.7		0.8 (0.4–1.5)
	2010–2020	20	5.7		1.0 (0.5–1.9)
Year of diagnosis	1989–2008 (older study)		6.0	0.716	1
	2008–2020 (recent study)	39	6.0		1.2 (0.7–2.1)

Bold values identify significant results with *p* ≤ 0.05 and confidence interval not including 1

p ≤0.05 = statitical significant, CI= Confidence Interval, R0 = Complete resection with clear margins, R1 = Resection with positive microscopic margins, R2 = Resection with positive macroscopic margins,

RT = Radiotherapy, CTx = Chemotherapy

Patients without distant metastases had a median OS of 8 months compared to the median OS of 4 months in patients with metastases.

When any kind of surgery was performed, an extension of OS from 3.6 to 9.8 months was observed, although it is questionable whether this was an effect of the surgery itself or of selection. If resection status R0 (clear margins) was reached, OS was better (14 months for R0 compared to 6 months for R +).

The median survival time for patients who received a total radiation dose of 50 Gy or higher was 13 months, versus 2.5 months in the comparison group (< 50 Gy).

In addition, a multimodal therapy consisting of surgery, radiotherapy and chemotherapy was associated with longer survival (9.8 months), compared to the use of only one or two therapy modalities. The median survival for use of surgery plus radiotherapy was 6 months, for radiation plus chemotherapy 4 months and for only radiotherapy 3 months.

Chemotherapy or concurrent radiochemotherapy had no significant effect on survival time, neither for patients with M0, nor for cases without metastases at the time of diagnosis. Likewise, absence of lymph node metastases, lymph node dissection, radiation delivery technique (IMRT or VMAT vs. 3D-konformal/opposing field technique) or histological differences (ATC vs. ATC + DTC) could not show any significant effect on OS.

**Fig. 3:**
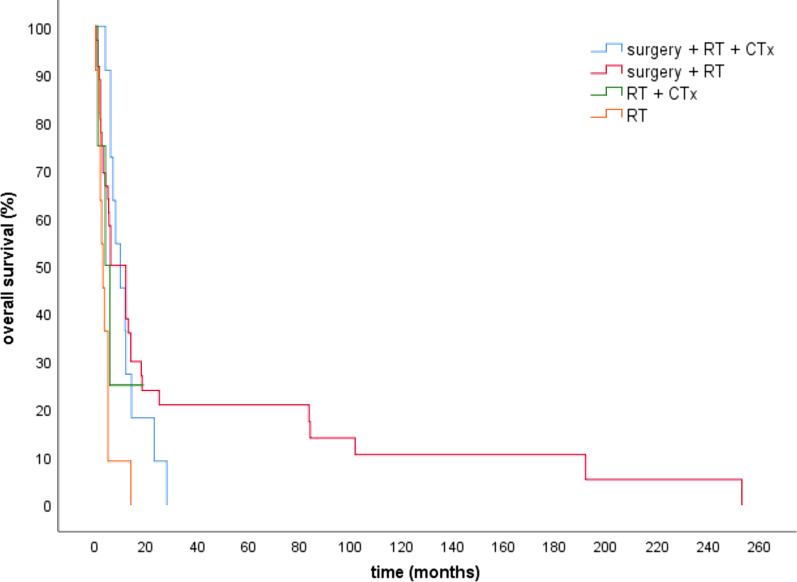
Overall survival for patients diagnosed until 2008 (older study) versus since 2008 (recent study)

There was no significant difference in overall survival depending on the year of diagnosis (Table 2, Fig. 2F). We observed the overall survival from the beginning until the end of the study in intervals of ten years. The median OS for patients whose ATC was diagnosed from 1989 to 1999 was 6 months as well as patients who were diagnosed between 2010 and 2020. Patients diagnosed from 2000 to 2009 lived on average 11.7 months. The median OS in patients of our older study was six months [5] as well as in the recent cohort (Fig. 3).

### Cox regression

A radiation dose of 50 Gy or higher appeared as the best prognostic factor with a HR of 0.3 (95% CI: 0.2–0.5), followed by surgery (HR = 0.4, 95% CI: 0.2–0.8) (Table 3). In a model comparing all the therapeutic options, adjusted for age, sex and Karnofsky performance scale, a higher radiation dose appeared as the only predictive factor for longer survival (HR = 0.2, 95% CI: 0.03–0.9). Chemotherapy had no significant effect on OS.

**Table 3:** Survival analysis comparing therapy modalities using cox proportional hazard models

Parameter	Univariate analysis (n = 63) HR (95% CI)	Model 1 (n = 63) HR (95% CI)	Model 2 (n = 21) HR (95% CI)	Model 3 (n = 21) HR (95% CI)
*Surgery*				
Yes	0.4 (0.2–0.8) *	0.4 (0.2–0.8) *		0.6 (0.2–1.6)
No	1	1	0.6 (0.3–1.6)	1
*Chemotherapy*				
Yes	1.0 (0.6–1.9)	1.0 (0.5–1.9)	0.9 (0.4–2.2)	0.7 (0.3–1.7)
No	1	1	1	1
*Radiochemotherapy (combined)*				
Yes	1.0 (0.5–1.9)	0.9 (0.4–1.9)	0.9 (0.4–2.2)	
No	1	1	1	
*Radiation dose*				
≥ 50 Gy	0.3 (0.2–0.5) *	0.3 (0.1–0.5) *	0.2 (0.03–1.1) **	0.2 (0.03–0.9) *
< 50 Gy	1	1	1	1

Bold values identify significant results with *p* ≤ 0.05 and confidence interval not including 1

Modell 1: Multivariate analysis with age and sex

Modell 2: Multivariate analysis with age, sex and Karnofsky scale

Modell 3: Multivariate analysis with surgery, chemotherapy, radiation dose, age, sex, Karnofsky scale

R = Hazard Ratio, CI = Confidence Interval

*p≤ 0.05 = statistical significant, **p = 0.05–0.1

### Stage IVC patients

In patients with distant metastases (stage IVC) neither surgery nor chemotherapy had a significant effect on OS, but again the higher radiation dose retained its prognostic significance in the univariate analysis (HR = 0.3, 95% CI: 0.1–0.9) when adjusted for age, sex and Karnofsky performance score and in the model with all therapy options (HR = 0.04, 95% CI: 0.002–0.7) (Table 4).

**Table 4:** Survival analysis comparing therapy modalities in patients with metastases at time of diagnosis using cox proportional hazard models

Parameter	Univariate analysis (n = 28) HR (95% CI)	Model 1 (n = 28) HR (95% CI)	Model 2 (n = 28) HR (96% CI)	Model 3 (n = 28) HR (95% CI)
*Surgery*				
Yes	0.9 (0.4–2.0)	0.9 (0.4–2.0)	1.6 (0.5–5.0)	1.6 (0.4–5.9)
No	1	1	1	1
*Chemotherapy*				
Yes	0.5 (0.2–1.4)	0.5 (0.2–1.4)	0.5 (0.1–1.6)	0.2 (0.04–1.2)
No	1	1	1	1
*Radiation Dose*				
≥ 50 Gy	0.3 (0.1–0.9)*	0.3 (0.1–1.1)	0.1 (0.01–1.1)	0.03 (0.002–0.7)*
< 50 Gy	1	1	1	1

Bold values identify significant results with *p* ≤ 0.05 and confidence interval not including 1

Modell 1: Multivariate analysis with age and sex

Modell 2: Multivariate analysis with age, sex and Karnofsky scale

Modell 3: Multivariate analysis with surgery, chemotherapy, radiation dose, age, sex, Karnofsky scale

*p ≤ 0.05 = statistical significant, ** *p*= 0.05–0.1

HR = Hazard ratio, CI = Confidence Interval

### Long-time survivors

In our patient collective, there were ten patients who lived longer than two years. These mostly had no distant metastases, only one had metastases at diagnosis and two other patients developed metastases in the course of the treatment.

All of these patients underwent surgery, three with complete resection and five with R2, for two patients the resection status was unknown. All long-time survivors received radiotherapy except one. The median total dose was 62.7 Gy.

### Acute toxicities due to radiation

Reported acute toxicities were newly developed, with radiation associated dysphagia and skin toxicity. Analyses with logistic regression showed a lower risk for severe skin toxicity using IMRT or VMAT (OR = 0.2, 95% CI: 0.04–0.9) compared to conventional radiation (3D conformal or opposing field technique). (Table 5).

**Table 5:** Analysis of acute toxicities using logistic regression models

Factor	Skin toxicity		Dysphagia	
	OR (95% CI)	*p*	OR (95% CI)	*p*
*Radiation dose*				
≥ 50 Gy	3.0 (0.9–10.4)	0.083	**0.2 (0.05–0.9)**	**0.029**
< 50 Gy	1		**1**	
*Radiation delivery technique*				
IMRT/VMAT	**0.2 (0.04–0.9)**	**0.045**	1.1 (0.2–4.7)	0.939
Conventional (3D/Opposing field)	**1**		1	
*Multimodal therapy*				
Yes	3.5 (0.4–29.1)	0.245	1.1 (0.2–7.5)	0.914
No	1		1	

Bold values identify significant results with *p* ≤ 0.05 and confidence interval not including 1

OR = Odds Radio, CI = Confidence Interval, IMRT = Intensity Modulated Radiotherapy. VMAT = Volumetric Modulated Arc Therapy, p ≤ 0.05 = statistical significant

The risk of dysphagia was lower if a radiation dose of at least 50 Gy was applied (OR = 0.2, 95% CI: 0.05–0.9).

Multimodal therapy (combination of surgery, chemotherapy and radiotherapy) failed to show any significant effect on skin toxicity or dysphagia.

## Discussion

In our analysis, we found five predictors for longer survival: the absence of distant metastasis at the time of diagnosis, surgery, complete resection (R0), a radiation dose of 50 Gy or higher and multimodal therapy (surgery, radiotherapy and chemotherapy). According to multivariate analyses, the best predictor of survival was a high radiation dose (≥ 50 Gy). Besides, even for patients with stage IV C (distant metastases) a radiation dose of at least 50 Gy could provide longer survival, whereas surgery and chemotherapy did not.

The guidelines of the National Comprehensive Cancer Network (NCCN) as well as the ATA (American Thyroid Association) guidelines recommend surgery (total thyroidectomy with therapeutic lymph node dissection), if resectable, with adjuvant radiotherapy and optional (radiosensitizing) chemotherapy for stage IVA and IVB [3, 8]. For unresectable disease they recommend radiotherapy and chemotherapy or molecularly targeted neoadjuvant therapy for borderline resectable disease. Depending on the response, surgery may follow. For stage IVC the guidelines distinguish aggressive from palliative treatment.

In the NCCN guideline aggressive treatment consists of surgery (total thyreoidectomy + lymphnode dissection, if resectable), locoregional radiation and chemotherapy plus molecular testing to check if a targeted therapy, such as dabrafenib/trametinib is considered [3]. Clinical trials should be initiated. Palliative therapy includes locoregional radiation as well as surgery and radiation of metastases. Tracheostomy and best supportive care may need to be performed as palliation or in transition before radiochemotherapy demonstrates effects. Following, cross-sectional imaging (CT, MRI with contrast), FDG PET/CT and disease monitoring should be continued.

The ATA guidelines focuse less on surgery and more on palliative cytotoxic chemotherapy and/or radiation for aggressive therapy in stage IVC disease [8]. They point out the importance of molecular testing and targeted therapies, already at the time of diagnosis.

In our study, a radiation dose of at least 50 Gy showed the strongest association with an improved survival, in accordance with the literature [9, 10]. However, an even more aggressive radiotherapy with a radiation dose of 60 Gy or higher should be delivered. In an analysis of 1288 ATC patients from the NCDB database, Pezzi et al. observed a significant benefit in survival for patients who received 60–75 Gy (HR = 0.419, 95% CI: 0.339–0.517) compared to cases who received 45–59.9 Gy (HR = 0.596, 95% CI:  0.479–0.743) and < 45 Gy (HR = 0.843, CI: 0.718–0.988) [11]. Comparable results can be found in other studies [2, 12, 13]. The NCCN guidelines also suggest a radiation dose of 60–66 Gy for adjuvant radiotherapy [3].

We also aimed to explore the difference in survival time and toxicity according to the radiation method. The advantage of IMRT and VMAT lies in the fact that a higher radiation dose can be delivered to the tumor while surrounding normal tissue can be better spared when compared to 3D-conformal techniques [2, 14]. Moreover, toxic side effects such as xerostomia can be reduced [15]. In their study on 41 ATC patients, Park et al. demonstrated a longer overall survival and progression-free survival using IMRT in comparison to 3D-conformal technique (HR for IMRT = 0.30) [2]. In addition, they could deliver a significant higher radiation dose using IMRT (66 vs. 60 Gy, *p* = 0.005), while toxicities were fewer than when compared to 3D-conformal technique.

In our cohort, a significantly higher radiation dose of 47.5 Gy versus 41.5 Gy could be delivered when using IMRT or VMAT. The toxic side effect of radiation was significantly better when IMRT or VMAT was used. Conversely, the risk of dysphagia was lower when a higher radiation dose was applied. This finding may be explained by the use of more toxic higher single doses (hypofractionation) in the radiotherapy concepts with lower total doses. Also, the OS was not improved using VMAT or IMRT compared to conventional radiotherapy. However, it must be pointed out that just a small number of our patients (13 out of 63) received IMRT or VMAT.

Moreover, surgery was a significant predictor for longer OS. Likewise, in almost every study we found, any kind of surgery extended overall survival and progression-free survival significantly, whereas radical surgery shows better outcomes [16–19].

For chemotherapy, we could not find any significant benefit in terms of survival. In the literature, various, but mostly disappointing outcomes, can be found [10, 20, 21].

On the other hand, regarding multimodal therapy, which includes chemotherapy as well as surgery and radiotherapy, we found a significant effect on survival, in accordance with the literature [2, 17, 22, 23]. Fan et al. explored the OS and progression-free survival (locoregional and distant) in 104 patients. Trimodal therapy was associated with an improved locoregional progression-free survival (HR = 0.060, *p* = 0.017) [13].

In our observation there was no advantage of survival in the most recent treatment periods (Table 2). The median OS in the last 10 years was six months, as well as in the period from 1989 until 1999. Compared to our older study from 2008, there was no difference in survival time (Fig. 3) [6].

Still, over the past years, treatment options have improved. Because of the high rate of BRAF V600E mutations in ATC recent advances have suggested the use of BRAF inhibitors, like dabrafenib and MEK inhibitors, such as trametinib [24].

In our cohort, three patients received targeted therapy. However, this number is too small and the observation period too short to draw any meaningful conclusions. The NCCN and ATA guidelines suggest dabrafenib plus trametinib for BRAF V600 mutated carcinoma, larotrectinib or entrectinib if NTRK gene fusion is positive and pralsetinib or selpercatinib for RET-fusion positive ATC [3, 8]. In case of a PD-21 expression or more than 10 mutations they also recommend checkpoint inhibitors such as pembrolizumab. Besides, the ATA guidelines name crizotinib and certinib for ALK-mutated tumors [8].

Several recent studies prove the positive effect of targeted therapy and immunotherapy [3, 25–31]. If there are mutations, which is the case in almost all the cases of ATCs, targeted therapies may be a promising therapeutic option or adjunct [3, 32]. The most frequent mutations are TP53, RAS and BRAF [32].

In our opinion, the future of ATC-therapy will be surgery plus individually planned high-dose radiotherapy and new targeted therapy. Even neoadjuvant concepts may become part of the therapeutic options in near future.

Our study was limited by the small number of patients. However, compared to other single center studies, 63 cases over 31 years still is a considerate number. Also, the results are biased by comorbidities or unresectable tumor which we tried to avoid by adjusting with Karnofsky score and building subgroups like the stage IVC group.

Due to the rareness of ATC prospective studies are very hard to perform. Hence, research is important to improve the therapy options and outcome of this extremely lethal disease.

## Conclusions

Based on our results, for treating ATC we could recommend surgery, if possible, with complete resection, radiotherapy with a radiation dose of 50 Gy or higher, and a multimodal therapy with surgery, radiation and chemotherapy. Compared to our previous results we could not see any significant advantage in overall survival [5]. But new radiation techniques (IMRT; VMAT) and targeted therapies could potentially improve the survival prognosis and reduce toxicity. More research is necessary to find new therapies and improve the outcome of this disease.

## Data Availability

The data presented in this study are available on request from the corresponding author in an anonymized form after data privacy check. The data are not publicly available due to data privacy regulations.

## Abbreviations

ATA: American Thyroid Association
ATC: Anaplastic thyroid cancer
CI: Confidence interval
CTCAE: Common terminology criteria of adverse events
CTx: Chemotherapy
Gy: Gray
HR: Hazard ratio
IMRT: Intensity modulated radiotherapy
NCCN: National Comprehensive Cancer Network
OS: Overall survival
R: Resection status
R0: Complete resection with clear margins
R1: Removal of all macroscopic disease, but positive microscopic margins
R2: Resection with macroscopic residual tumor
R +: Macroscopic or microscopic residual tumor R1 or R2
RX: Resection stage cannot be assessed
RT: Radiotherapy
UICC: Union for International Cancer Control
VMAT: Volumetric modulated arc radiotherapy

## References

[1] Tashima L, Mitzner R, Durvesh S, Goldenberg D (2012). Dyspnea as a prognostic factor in anaplastic thyroid carcinoma. Eur Arch Otorhinolaryngol.

[2] Park JW, Choi SH, Yoon HI, Lee J, Kim TH, Kim JW, Lee IJ (2018). Treatment outcomes of radiotherapy for anaplastic thyroid cancer. Radiat Oncol J.

[3] National Comprehensive Cancer Network (Ed.) (2021): NCCN Clinical Practice Guideline in Oncology (NCCN Guidelines). Thyroid Carcinoma. Version 3.2021. https://www.nccn.org/guidelines/category_1.

[4] Lin B, Ma H, Ma M, Zhang Z, Sun Z, Hsieh I (2019). The incidence and survival analysis for anaplastic thyroid cancer: a SEER database analysis. Am J Transl Res.

[5] Oweida A, Phan A, Vancourt B, Robin T, Hararah MK, Bhatia S (2018). Hypofractionated radiotherapy is superior to conventional fractionation in an orthotopic model of anaplastic thyroid cancer. Thyroid.

[6] Dumke A-K, Pelz T, Vordermark D (2014). Long-term results of radiotherapy in anaplastic thyroid cancer. Radiat Oncol.

[7] National Cancer Institute. Common Terminology Criteria for Adverse Events (CTCAE).

[8] Bible KC, Kebebew E, Brierley J, Brito JP, Cabanillas ME, Clark TJ (2021). 2021 American thyroid association guidelines for management of patients with anaplastic thyroid cancer. Thyroid.

[9] Tiedje V, Stuschke M, Weber F, Dralle H, Moss L, Führer D (2018). Anaplastic thyroid carcinoma: review of treatment protocols. Endocr Relat Cancer.

[10] Wendler J, Kroiss M, Gast K, Kreissl MC, Allelein S, Lichtenauer U (2016). Clinical presentation, treatment and outcome of anaplastic thyroid carcinoma: results of a multicenter study in Germany. Eur J Endocrinol.

[11] Pezzi TA, Mohamed ASR, Sheu T, Blanchard P, Sandulache VC, Lai SY (2017). Radiation therapy dose is associated with improved survival for unresected anaplastic thyroid carcinoma: Outcomes from the National Cancer Data Base. Cancer.

[12] Glaser SM, Mandish SF, Gill BS, Balasubramani GK, Clump DA, Beriwal S (2016). Anaplastic thyroid cancer: prognostic factors, patterns of care, and overall survival. Head Neck.

[13] Fan D, Ma J, Bell AC, Groen AH, Olsen KS, Lok BH (2020). Outcomes of multimodal therapy in a large series of patients with anaplastic thyroid cancer. Cancer.

[14] Evers C, Ostheimer C, Sieker F, Vordermark D, Medenwald D (2020). Benefit from surgery with additional radiotherapy in N1 head and neck cancer at the time of IMRT: a population-based study on recent developments. PLoS One..

[15] Nutting CM, Morden JP, Harrington KJ, Urbano TG, Bhide SA, Clark C (2011). Parotid-sparing intensity modulated versus conventional radiotherapy in head and neck cancer (PARSPORT): a phase 3 multicentre randomised controlled trial. Lancet Oncol.

[16] Huang N-S, Shi X, Lei B-W, Wei W-J, Lu Z-W, Yu P-C (2019). An update of the appropriate treatment strategies in anaplastic thyroid cancer: a population-based study of 735 patients. Int J Endocrinol.

[17] Salehian B, Liem SY, Mojazi Amiri H, Maghami E (2019). Clinical trials in management of anaplastic thyroid carcinoma; progressions and set backs: a systematic review. Int J Endocrinol Metab..

[18] Hu S, Helman SN, Hanly E, Likhterov I (2017). The role of surgery in anaplastic thyroid cancer: A systematic review. Am J Otolaryngol.

[19] Sugitani I, Hasegawa Y, Sugasawa M, Tori M, Higashiyama T, Miyazaki M (2014). Super-radical surgery for anaplastic thyroid carcinoma: a large cohort study using the Anaplastic Thyroid Carcinoma Research Consortium of Japan database. Head Neck.

[20] Xia Q, Wang W, Xu J, Chen X, Zhong Z, Sun C (2018). Evidence from an updated meta-analysis of the prognostic impacts of postoperative radiotherapy and chemotherapy in patients with anaplastic thyroid carcinoma. Onco Targets Ther.

[21] Mohebati A, Dilorenzo M, Palmer F, Patel SG, Pfister D, Lee N (2014). Anaplastic thyroid carcinoma: a 25-year single-institution experience. Ann Surg Oncol.

[22] Rao SN, Zafereo M, Dadu R, Busaidy NL, Hess K, Cote GJ (2017). Patterns of treatment failure in anaplastic thyroid carcinoma. Thyroid.

[23] Haymart MR, Banerjee M, Yin H, Worden F, Griggs JJ (2013). Marginal treatment benefit in anaplastic thyroid cancer. Cancer.

[24] Agrawal VR, Hreno J, Patil T, Bowles DW (2018). New therapies for anaplastic thyroid cancer. Drugs Today.

[25] Park J, Jung HA, Shim JH, Park W-Y, Kim TH, Lee S-H (2021). Multimodal treatments and outcomes for anaplastic thyroid cancer before and after tyrosine kinase inhibitor therapy: a real-world experience. Eur J Endocrinol.

[26] Capdevila J, Wirth LJ, Ernst T, Aix SP, Lin CC, Ramlau R, Butler MO, Delord JP, Gelderblom H, Ascierto PA, Fasolo A, Führer D, Hütter-Krönke ML, Forde PM, Wrona A, Santoro A, Sadow PM, Szpakowski S, Wu H, Bostel G, Faris J, Cameron S, Varga A, Taylor M (2020). PD-1 Blockade in anaplastic thyroid carcinoma. J Clin Oncol.

[27] Tahara M, Kiyota N, Yamazaki T, Chayahara N, Nakano K, Inagaki L (2017). Lenvatinib for anaplastic thyroid cancer. Front Oncol.

[28] Wächter S, Wunderlich A, Roth S, Mintziras I, Maurer E, Hoffmann S (2018). Individualised multimodal treatment strategies for anaplastic and poorly differentiated thyroid cancer. J Clin Med.

[29] Lim AM, Solomon BJ (2020). Immunotherapy for anaplastic thyroid carcinoma. J Clin Oncol.

[30] Ito Y, Onoda N, Ito K-I, Sugitani I, Takahashi S, Yamaguchi I (2017). Sorafenib in Japanese patients with locally advanced or metastatic medullary thyroid carcinoma and anaplastic thyroid carcinoma. Thyroid.

[31] Robb R, Yang L, Shen C, Wolfe AR, Webb A, Zhang X (2019). Inhibiting BRAF oncogene-mediated radioresistance effectively radiosensitizes BRAFV600E-mutant thyroid cancer cells by constraining DNA double-strand break repair. Clin Cancer Res.

[32] Bonhomme B, Godbert Y, Perot G, Al Ghuzlan A, Bardet S, Belleannée G (2017). Molecular pathology of anaplastic thyroid carcinomas: a retrospective study of 144 cases. Thyroid.

